# Jejunoileal mucosal growth in mice with a limited microbiome

**DOI:** 10.1371/journal.pone.0266251

**Published:** 2022-03-29

**Authors:** Matthew P. Shaughnessy, Christine J. Park, Pooja S. Salvi, Robert A. Cowles

**Affiliations:** Division of Pediatric Surgery, Department of Surgery, Yale University, New Haven, Connecticut, United States of America; INRAE, FRANCE

## Abstract

Previous work demonstrated enhanced enterocyte proliferation and mucosal growth in gnotobiotic mice, suggesting that intestinal flora participate in mucosal homeostasis. Furthermore, broad-spectrum enteral antibiotics are known to induce near germ-free (GF) conditions in mice with conventional flora (CONV). We hypothesized that inducing near GF conditions with broad-spectrum enteral antibiotics would cause ordered small intestinal mucosal growth in CONV mice but would have no effect in GF mice with no inherent microbiome. C57BL/6J CONV and GF mice received either an antibiotic solution (Ampicillin, Ciprofloxacin, Metronidazole, Vancomycin, Meropenem) or a vehicle alone. After treatment, small intestinal villus height (VH), crypt depth (CD), mucosal surface area (MSA), crypt proliferation index (CPI), apoptosis, and villus and crypt cell types were assessed. Antibiotic-treated CONV (Abx-CONV) mice had taller villi, deeper crypts, increased CPI, increased apoptosis, and greater MSA compared to vehicle-treated CONV mice. Minor differences were noted in enterocyte and enterochromaffin cell proportions between groups, but goblet and Paneth cell proportions were unchanged in Abx-CONV mice compared to vehicle-treated CONV mice (p>0.05). Antibiotics caused no significant changes in VH or MSA in GF mice when compared to vehicle-treated GF mice (p>0.05). Enteral administration of broad-spectrum antibiotics to mice with a conventional microbiome stimulates ordered small intestinal mucosal growth. Mucosal growth was not seen in germ-free mice treated with antibiotics, implying that intestinal mucosal growth is associated with change in the microbiome in this model.

## Introduction

Intestinal failure (IF), most commonly in the form of short bowel syndrome (SBS), is a complex and morbid clinical condition that results in the inability to maintain fluid, electrolyte, and nutritional balances necessary to sustain life [1]. SBS occurs following extensive surgical resection of small intestine and, following massive small bowel resection, the intestine is known to undergo compensatory changes that increase absorptive capacity in the setting of reduced functional mass. This process, known as intestinal adaptation, is characterized by increased villus height (VH), crypt depth (CD), and crypt cell proliferation [2,3]. Many investigators have attempted to mimic intestinal adaptation for therapeutic benefit whether through pharmaceuticals targeting intestinal growth factor receptors and neurotransmitters or through dietary alterations [4,5]. By promoting intestinal growth, these therapies may help rehabilitate the remaining small intestine in patients with SBS. To assess mucosal adaptive growth, investigators often analyze anatomic and histologic parameters of VH, CD, and mucosal surface area (MSA) as surrogate measures of mucosal absorption and function. The present study aims to evaluate a novel potential target involved in small intestinal homeostasis, namely the small intestinal microbiome.

The small intestinal microbiome is a vast and heterogenous population of non-pathogenic microbes ranging in population density from 10^3^–10^8^ CFU/ml [6]. The commensal microbiome is known to participate in the host immune response, metabolism, intestinal development, and intestinal homeostasis [7–9]. Although the specific function of these microbial populations remains under investigation in the healthy host, the microbiome has already been implicated in various disease states [9]. Not surprisingly, the intestinal microbiome is markedly altered in patients with SBS, and often demonstrates an imbalance between pro- and anti-inflammatory commensal bacteria [10,11]. In fact, some have found that an overabundance of certain *Enterobacteriaceae* is associated with an inability to wean from parenteral nutrition, suggesting the potential for therapeutic approaches aimed at targeting specific intestinal microbes in patients with SBS [12,13].

A popular paradigm for the study of the intestinal microbiome relies on the use of germ-free animals. The germ-free condition, characterized by a complete absence of living organisms, and thus no inherent intestinal microbiome, serves as a valid experimental model for the study of host-microbial interactions in both health and disease [14]. Not surprisingly, the intestinal mucosa of germ-free animals is different morphologically from the mucosa of animals with conventional flora. While the specifics of these differences are controversial, early studies suggested that the germ-free condition may be associated with taller, narrower villi in the proximal small intestine [15–17]. Given these findings, investigators have surmised that there may be a mucosal benefit to the germ-free condition as it pertains to intestinal adaptation following small bowel resection. In fact, the germ-free condition results in a more robust adaptive response following small bowel resection in rats, suggesting that the presence of intestinal flora may diminish the adaptive response in the host [18]. These findings inspired previous work aimed at investigating the intestinal mucosal changes observed in a setting of dramatically narrowed small intestinal microbiome in gnotobiotic mice [19]. Gnotobiotic mice, colonized with a single bacterial organism, demonstrated significant increases in intestinal morphometric growth parameters in comparison to mice with conventional flora [19]. In fact, all gnotobiotic mice studied had taller villi than mice with conventional flora, regardless of the bacterial strain colonizing the gnotobiotic mouse. Combined, these findings suggest that germ-free or near germ-free states may provide optimal conditions to promote intestinal mucosal growth. As neither the germ-free nor the gnotobiotic condition is reproducible in human studies, however, more clinically translatable approaches for studying the mucosal effects of alterations within the intestinal microbiome are needed. One method of study utilizes the administration of broad-spectrum enteral antibiotics, through a process termed antibiotic-induced microbiome depletion (AIMD) [20,21]. AIMD models allow investigators to determine the physiologic changes associated with a markedly depleted intestinal microbiome in a host that begins with an otherwise unperturbed microbiome.

In an attempt to find a clinical correlate to the findings demonstrated in germ-free rats and gnotobiotic mice [18,19], the present study investigates the intestinal mucosal effects of an AIMD regimen of broad spectrum enteral antibiotics known to induce near germ-free conditions in mice [22–24]. Such near germ-free conditions may have the potential to replicate the mucosal changes observed in gnotobiotic mice. It is important to note that other studies employing AIMD have revealed the potential for microbiome-independent antibiotic effects on the host intestine [25]. Thus, in order to implicate the microbiome depletion as integral to the adaptive process, we examined the mucosa of both conventional flora mice and germ-free mice treated with the same antibiotic regimen. Ultimately, we hypothesized that limiting the intestinal microbiome with antibiotics would result in ordered small intestinal mucosal growth in mice that originally harbored conventional flora (CONV) but similar treatment would have no effect on the mucosa of germ-free (GF) mice with inherently absent intestinal flora.

## Results

### Metabolic data

**Fig 1:**
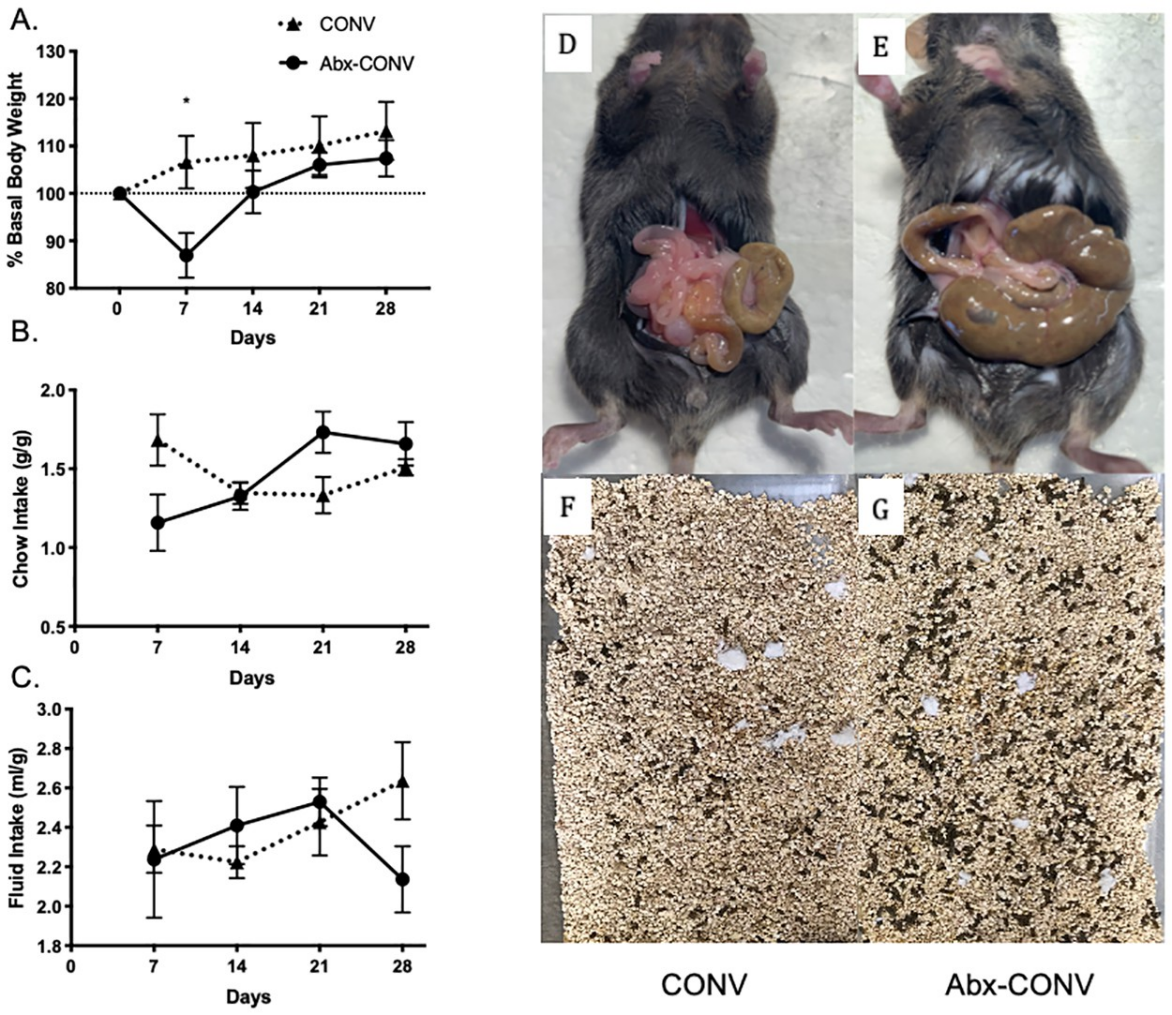
Metabolic measurements and gross findings in CONV (n = 4) and Abx-CONV (n = 5) mice after 4 weeks of treatment. (A) Abx-CONV mice demonstrated a period of % basal body weight loss (gram experimental day / gram initial x100) at 7 days, which was subsequently gained back at day 14 and sustained through day 28. (B,C) There were no significant differences in fluid intake (ml/g) or chow intake (g/g) between the groups. Gross findings at time of laparotomy at day 28 comparing vehicle (D) and Abx-CF (E). Abx-CF mice demonstrated a classic appearance of a dilated, stool filled cecum consistent with near germ-free conditions. One-week stool production from day 21 to day 28 was notably increased in Abx-CF mice (G) compared to vehicle (F). Values presented as Mean±SEM, *p<0.05.

The metabolic effects of the antibiotic regimen were evaluated over a 28-day treatment course in CONV mice. After 7-days, antibiotic-treated CONV mice (Abx-CONV) demonstrated a significant decrease in percent basal body weight when compared to vehicle (p = 0.03, Fig 1A). Mice regained this weight by 14 days and demonstrated no further difference in percent basal body weight over the remaining treatment period when compared to control. No significant differences were observed in food intake or fluid intake by weight between groups (Fig 1B and 1C). Gross findings at time of laparotomy revealed markedly dilated and stool-filled ceca amongst Abx-CONV mice (Fig 1E) compared to vehicle (Fig 1D). These macroscopic findings were anticipated and have been shown by others to imply the presence of a near germ-free state [26]. This corresponded with a gross increase in the overall stool output amongst Abx-CONV mice (Fig 1G) compared to vehicle (Fig 1F).

### Microbiome analysis

**Fig 2:**
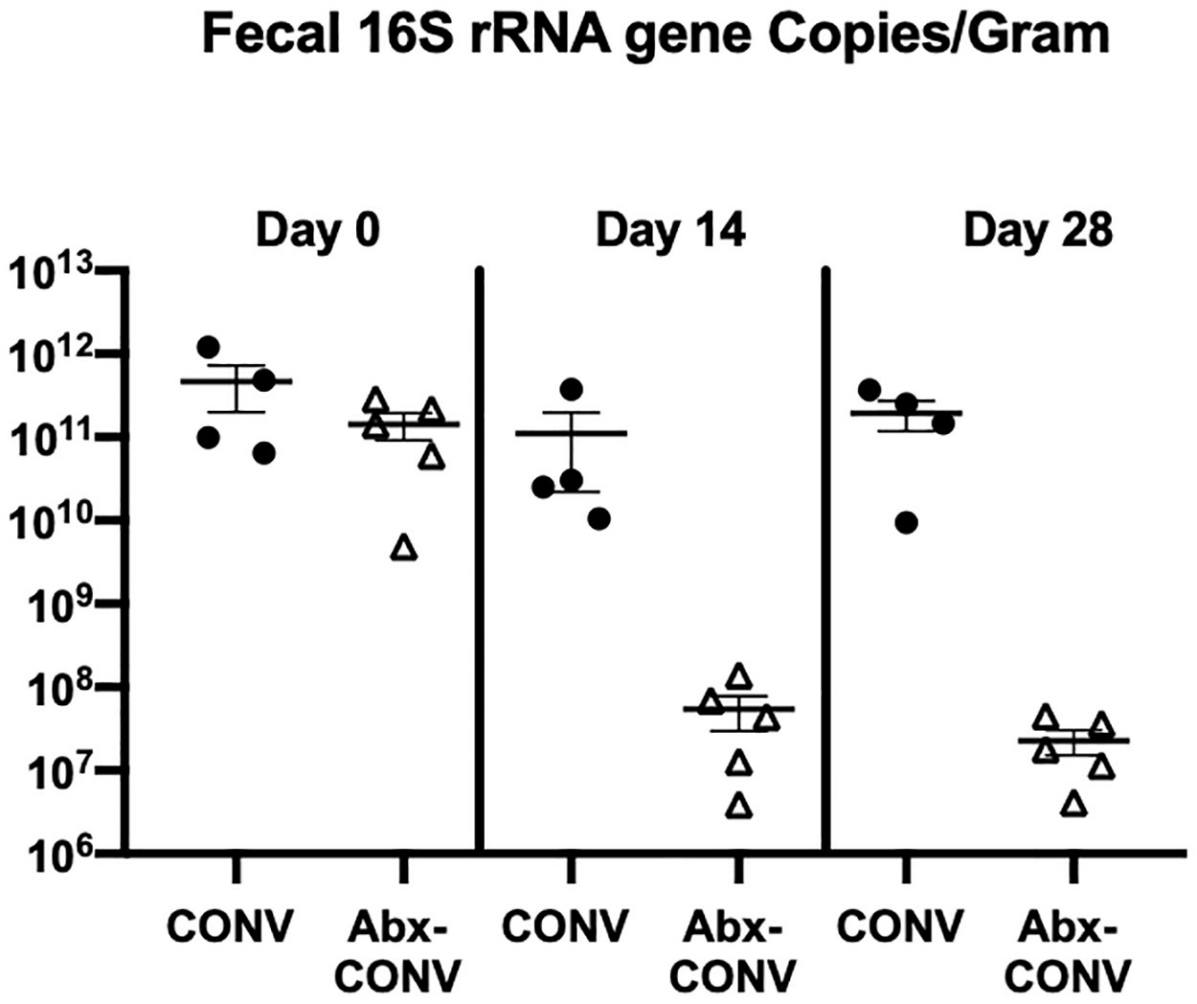
Microbiome analysis. Treatment with antibiotics resulted in a decrease in absolute quantification of 16S rRNA copies/gram at both day 14 and day 28 (n = 4 CONV mice, n = 5 Abx-CONV mice).

Microbial density of fecal pellets amongst CONV and Abx-CONV mice was calculated at time points of day 0, day 14, and day 28. In each case, the absolute quantification (16S copies/gram) of fecal stool pellets was calculated. Treatment with antibiotics resulted in a considerable decrease in absolute quantification of 16S rRNA copies/gram at both day 14 and day 28 (1.10e11 vs 5.41e7, p = 0.2; 1.94e11 vs 2.28e7, p = 0.02 respectively, Fig 2). There was no difference in absolute quantification of 16S rRNA between groups at day 0 (4.63e11 vs 1.41e11, p = 0.2).

### Morphometric parameters in conventional flora mice

**Fig 3:**
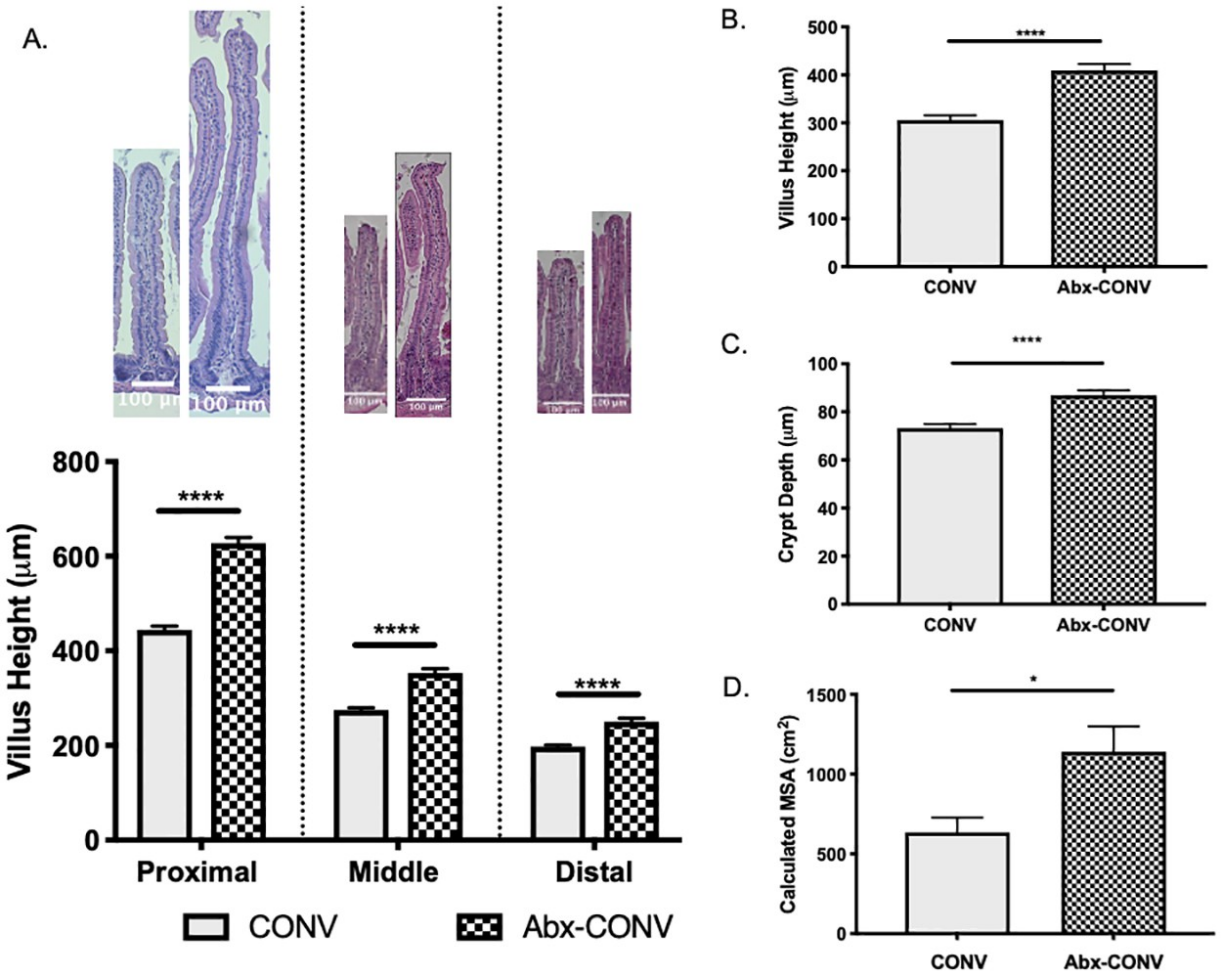
Morphometric parameters in CONV mice after 28 days of treatment. (A) Abx-CONV mice demonstrate considerably taller villi in the proximal, middle, and distal small intestine compared to vehicle as depicted histologically and graphically. (B) Mean villus height, (C) mean crypt depth, and (D) mean calculated mucosal surface area for all small intestinal regions are also significantly greater in the Abx-CONV mice. Values presented as Mean±SEM; *p<0.05, ****p<0.0001; n = 4 CONV mice, n = 5 Abx-CONV mice.

Abx-CONV mice demonstrated a significant increase in VH in the proximal, middle, and distal small intestine when compared to vehicle (p<0.0001 for all regions) at 28 days (Fig 3A). As such, the mean villus height for all small intestinal regions combined was also greater in Abx-CONV mice (409.3±13.2μm versus 305.7±10.1μm, p<0.0001, Fig 3B). Crypts were deeper in Abx-CONV mice as well (86.9±2μm versus 73.2±1.7μm, p<0.0001, Fig 3C). Mean MSA was calculated for each group and found to be greater in Abx-CONV mice (1141±158cm^2^ versus 634±99cm^2^, p = 0.03, Fig 3D).

**Fig 4:**
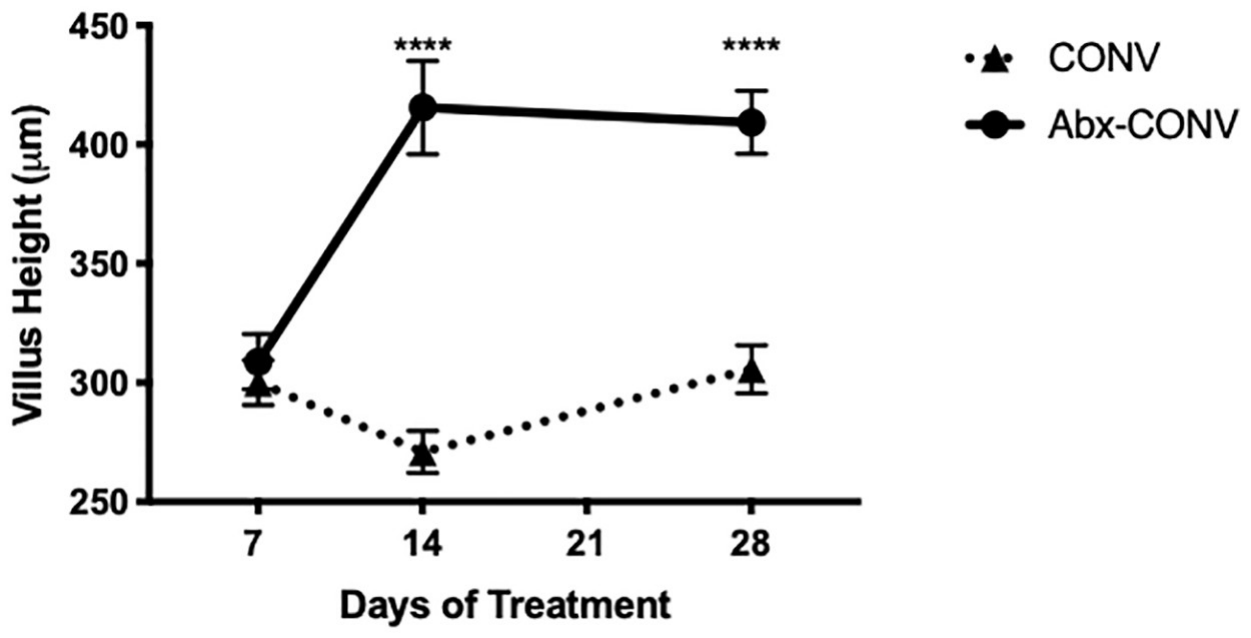
Optimal timing of antibiotic therapy necessary to induce intestinal mucosal growth. Abx-CONV mice were treated for a duration of 1 (n = 6), 2 (n = 6), and 4 (n = 9) weeks. Intestinal mucosal changes are observed only after 2–4 weeks of enteral antibiotic therapy. Values presented as Mean±SEM, ****p<0.0001.

The time course of the morphologic changes was assessed by treating mice with antibiotics for varying durations of 7, 14, and 28 days. There were no significant changes in VH, CD, or MSA after 7 days of antibiotic treatment. However, VH and MSA were both significantly greater in Abx-CONV mice after 14 days, mirroring the pattern demonstrated at 28 days (Fig 4). Given these findings, all additional studies were thus conducted over an optimized 14-day treatment period.

### Crypt proliferation index and apoptosis

**Fig 5:**
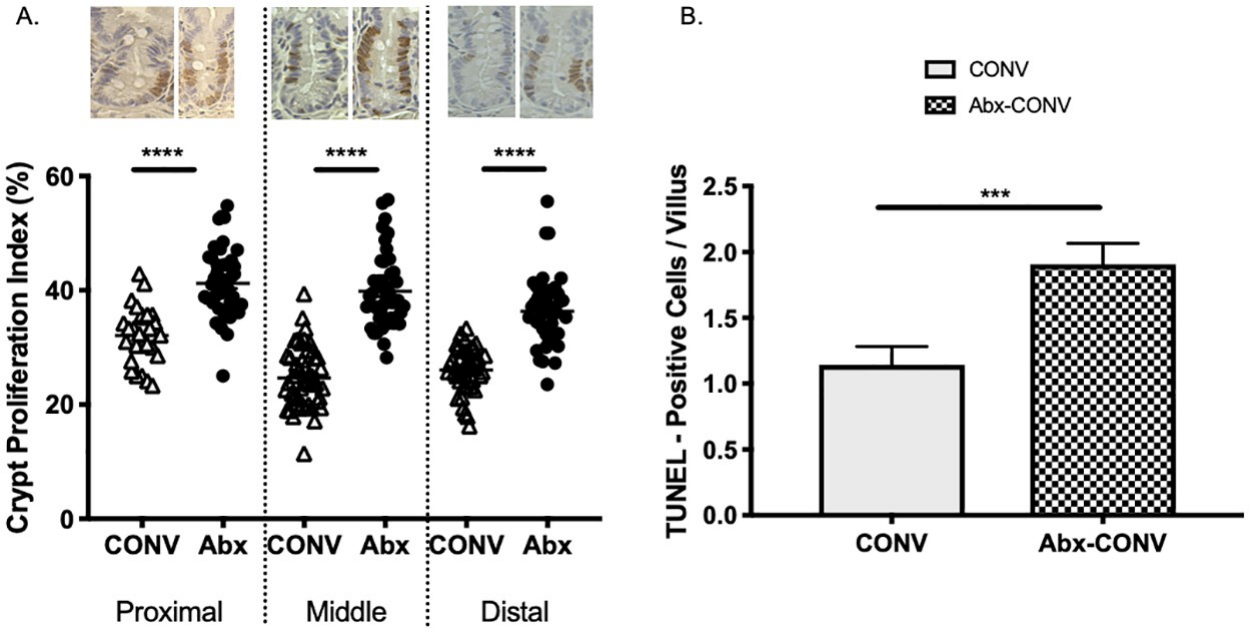
Crypt proliferation index (CPI, % BrdU positive cells / total crypt cells) and degree of apoptosis. (A) CPI is greater in Abx-CONV compared to vehicle in the proximal, middle, and distal small intestine with images representative of BrdU incorporation in crypts of each intestinal segment. (B) Apoptosis of enterocytes is greater in Abx-CONV mice. Values presented as Mean±SEM; ***p<0.001, ****p<0.0001; n = 3 CONV mice, n = 3 Abx-CONV mice.

Crypt Proliferation Index (CPI) was greater in the proximal, middle, and distal small intestine of Abx-CONV mice when compared to vehicle (Fig 5A). We also assessed the extent of villus apoptosis to determine if the increase in proliferation noted at the crypt level corresponded with villus cell turnover or if the increases in villus height could be explained by a blunted apoptotic activity. The TUNEL procedure was used to assess apoptosis in Abx-CONV and control mice. The total number of cells undergoing apoptosis was greater in Abx-CONV mice (Fig 5B).

### Cellular composition

**Fig 6:**
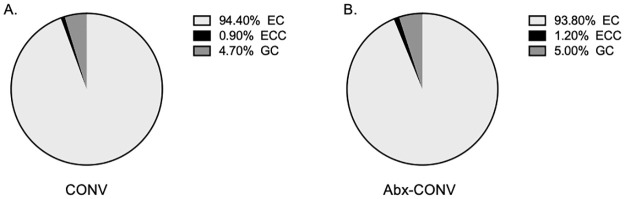
Cellular composition of intestinal epithelium in vehicle and Abx-CONV mice. The proportion of villus epithelial cells (% cell type / total nucleated cells) is similar between (A) Vehicle and (B) Abx-CONV. EC (Enterocyte), ECC (Enterochromaffin Cell), GC (Goblet Cell). n = 3 CONV mice, n = 3 Abx-CONV mice.

To test the hypothesis that antibiotic treatment stimulates mucosal growth that is ordered with an equal distribution of villus and crypt cell types between Abx-CONV and control mice, the intestinal mucosa was stained with immunofluorescent markers to identify villus and crypt cell subtypes. Abx-CONV mice had a significant increase in total number of cells per villus and an increase in the total number of each individual cell type along the entire small bowel (118.8±4.7 cells versus 95.1±2.8 cells, p<0.0001), representative of the overall increase in villus height. Individual cell types per villus were also increased with Abx-CONV mice having more Enterocytes (111.5±4.4 cells versus 87.3±2.7 cells, p<0.0001), Enterochromaffin cells (1.4±0.1 cells versus 0.8±0.1 cells, p = 0.0002), and Goblet cells (6±0.3 cells versus 4.4±0.2 cells, p<0.0001). There were no significant differences in the total number of Paneth cells per crypt between the groups with 4±0.1 Paneth cells per crypt in CONV mice versus 3.9±0.1 in Abx-CONV mice (p > 0.05). When analyzing cell type distribution in villi, there was no significant difference in the proportion of GCs per villus, however, there was a slight increase in the proportion of ECCs in the Abx-CONV mice (1.2% versus 0.9%. Fig 6). This corresponded with a similarly slight decrease in the proportion of ECs in the Abx-CONV mice (93.8% versus 94.4%).

### Serum citrulline

Serum citrulline measurements were obtained as a surrogate measure of functional enterocyte mass. Citrulline levels were obtained at 0, 14, and 28 days and presented as a change in serum concentration over time. Abx-CONV mice demonstrated a numerical increase in serum citrulline compared to vehicle (172.3μM versus -65.2μM, p = 0.09), corresponding with an increase in total EC per villus, although this did not reach statistical significance (p = 0.09; *n = 4 CONV mice*, *n = 5 Abx-CONV mice)*.

### Germ-free comparison

In order to test the hypothesis that the intestinal mucosal effects of the antibiotic regimen are secondary to alterations in the intestinal microbiome, we administered the antibiotic regimen to germ-free mice inherently lacking an intestinal microbiome. Antibiotic treated germ-free mice (Abx-GF) demonstrated no significant increase in VH, CD, or MSA when compared to control (V-GF, Table 1).

**Table 1:** Morphometric parameters in germ-free mice (Mean±SEM).

	V-GF	Abx-GF	p-value
**Villus Height (VH)**	411.3±10.6	406.9±10.4μm	0.77
**Crypt Depth (CD)**	78.9±1.1μm	80.1±1.1μm	0.41
**Mucosal Surface Area (MSA)**	1644±12cm^2^	1362±10cm^2^	0.09

*Mean values for all regions of sampled small intestine combined (n = 6 V-GF, n = 6 Abx-GF mice).

### Reversal of antibiotic therapy

**Fig 7:**
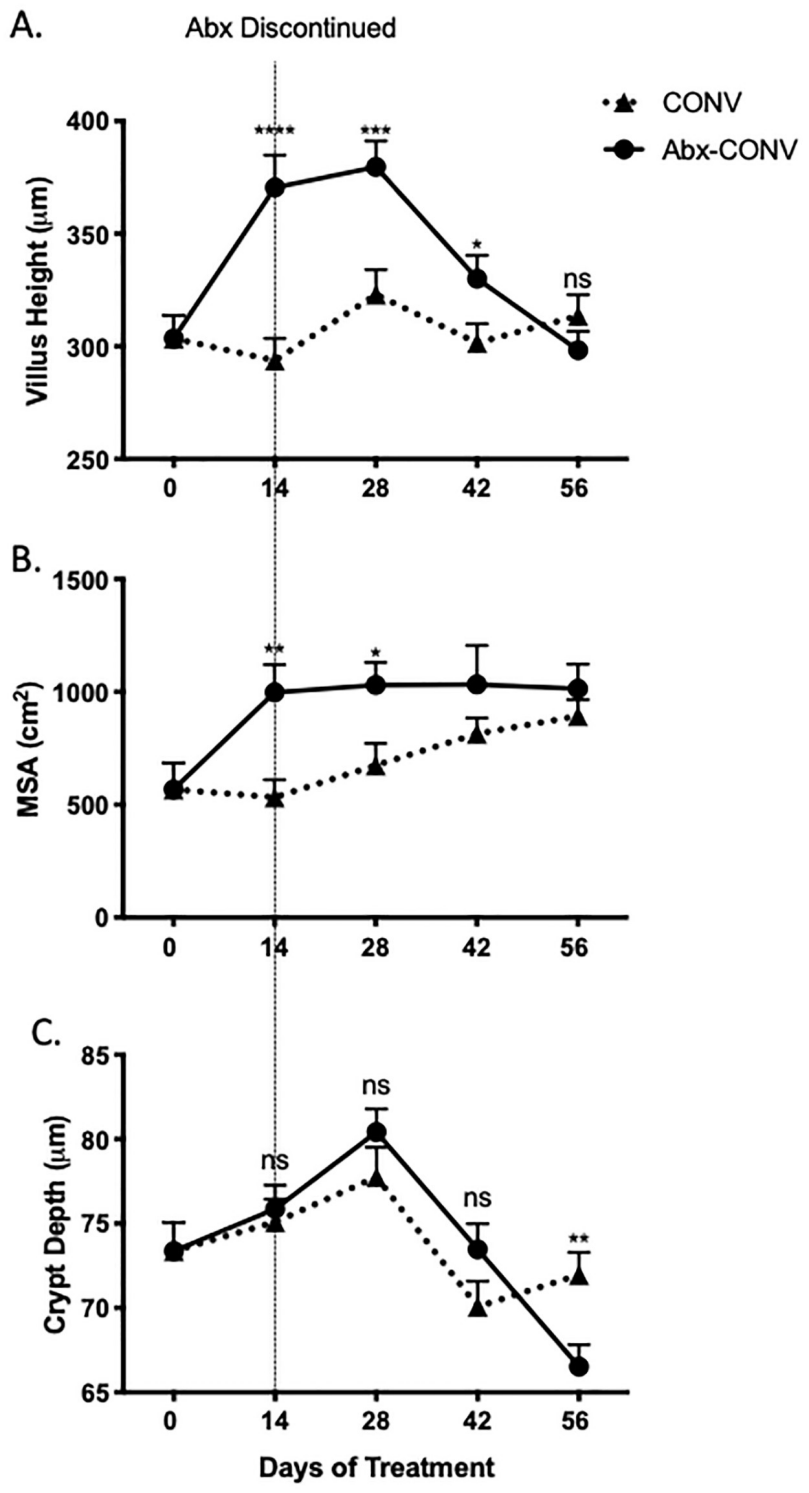
Cessation of antibiotic treatment and reversal of mucosal changes. (A) Antibiotic treated mice demonstrated significant increases in villus height after 14 days of antibiotic therapy, though returned to baseline by day 56. (B) Calculated mucosal surface area (MSA) was greater in antibiotic treated mice at day 14 of treatment and returned to baseline after cessation of antibiotics by day 42. (C) There were no significant (ns) difference noted in crypt depth between the two groups. Values presented as Mean±SEM; *p<0.05, ** p<0.01, ***p<0.001, ****p<0.0001; n = 12 mice.

We next aimed to test if the effects of antibiotics on the intestinal mucosa were permanent or reversible. As had been seen in earlier experiments, antibiotic treated mice demonstrated significant increases in villus height after 14 days of antibiotic therapy, an effect which persisted for an additional 28 days after antibiotic therapy though eventually returned to baseline by day 56 (Fig 7A). MSA was also increased in antibiotic treated CONV mice at day 14, persisting for an additional 14 days after antibiotic cessation and returning to baseline by day 42. (Fig 7B). Crypts were deeper in Abx mice at day 14, 28, and 42, although this did not reach statistical significance (Fig 7C).

### Narrowing the antibiotic regimen

**Fig 8:**
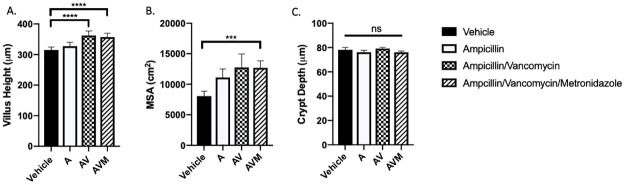
Use of narrower antibiotic regimens. Demonstration of morphometric parameters in CONV mice after 14 days of treatment with either Vehicle, Ampicillin (A), Ampicillin/Vancomycin (AV), or Ampicillin/Vancomycin/Metronidazole (AVM). (A) Combined villus height is increased in both AV and AVM treated mice. (B) Calculated mucosal surface area (MSA) was increased in AVM treated mice compared to vehicle. (C) No significant (ns) difference was noted in crypt depth between the groups. Values presented as Mean±SEM; ***p<0.001, ****p<0.0001; n = 4 CONV mice, n = 4 A mice, n = 4 AV mice, n = 4 AVM mice.

Mice were treated with a variety of antibiotic combinations in an effort to promote intestinal mucosal growth with a less toxic side effect profile. After 14 days of treatment, there were no significant differences in food intake, fluid intake, or weight gain or loss between groups. Mice undergoing treatment with Ampicillin (A), Ampicillin/Vancomycin (AV), and Ampicillin/Vancomycin/Metronidazole exhibited VH increases in the proximal and middle small intestine when compared to vehicle (*p*<0.001). No significant increases were noted in VH in the distal small intestine for any of the limited antibiotic regimens. When VH measurements from all regions were combined, treatment with AV and AVM caused increased VH compared to control (Fig 8A), while VH after treatment with A alone was no different from vehicle. AVM treatment was the only limited antibiotic regimen to result in an increased MSA compared to control (Fig 8B). None of the limited antibiotic regimens resulted in increased CD (Fig 8C).

## Discussion

The present study investigated the morphologic changes in the small intestinal mucosa following broad spectrum enteral antibiotic therapy (Ampicillin, Ciprofloxacin, Metronidazole, Vancomycin, and Meropenem) derived from other regimens known to markedly reduce the intestinal microbiome and approach near GF conditions in mice with conventional flora [22–24]. Antibiotic treatment resulted in dramatic microbial depletion at both fourteen days and twenty-eight days of treatment. After fourteen to twenty-eight days of antibiotic therapy, Abx-CONV mice demonstrated marked intestinal mucosal growth with marked increases in VH, CD, and MSA throughout the small intestine. In addition, antibiotic therapy in mice with conventional flora resulted in increased proliferative indices (CPI) and a corresponding increase in overall enterocyte apoptosis. After cessation of antibiotic treatment, mucosal growth did not immediately reverse but persisted for 14 to 28 days following therapy, suggesting that there is a lasting effect on mucosal proliferation even as the intestinal microbiome recovers.

Given the profound intestinal mucosal growth following antibiotic therapy, we further aimed to determine if the villi and crypts displayed normal proportions of cellular types. An analysis of cellular composition revealed a similar distribution of villus and crypt cell types between the treatment groups suggesting that the mucosal changes with antibiotic treatment were in fact ordered as opposed to neoplastic or asymmetric toward one cell type of another. Furthermore, there was no observable shift toward a particular non-absorptive cell type such as goblet cells or enteroendocrine cells. Importantly, the treatment preserved the composition of Paneth cells and goblet cells, both known to be essential for their role in enteric innate immune response [27,28].

When a cohort of GF mice, with no detectable colonization of intestinal flora, was treated with this broad-spectrum antibiotic regimen there were no observable increases in VH, CD, or MSA. When taken together, these findings suggest that enteral antibiotic therapy has no direct pharmacologic, metabolic, or physiologic effect in the absence of a microbiome and thus relies upon the alteration or reduction of the native intestinal microbiome in order to induce lasting and ordered intestinal mucosal growth in mice.

The data presented also suggest that the observed histologic changes were most notable within the proximal small intestine, where villus height and mucosal surface area were substantially greater in antibiotic treated mice. This is consistent with most of the original findings in germ-free animals, which suggested that the proximal small intestine of germ-free mice have taller and more narrow villi than that of their conventional flora counterparts [19–21]. One explanation for these findings is the relatively low density of indigenous bacteria within the proximal small intestine compared to the terminal ileum [6]. This may be representative of a more complete elimination of the intestinal microbiome in these regions, allowing for a closer representation of the intestinal mucosa in germ-free mice. Traditionally, the terminal ileum is largely regarded as the most responsive to adaptation following small bowel resection and has been the focus of many previous targeted therapies [29,30]. This makes the antibiotic model appealing to be used in conjunction with other treatment modalities that may be more effective at targeting mucosal growth in the terminal ileum. Future study is warranted to determine the effects of enteral antibiotic therapy on microbial population density at specific gastrointestinal locations in addition to that of fecal samples.

This is not the first study to suggest that treatment with enteral antibiotics causes intestinal mucosal changes. The livestock industry has long utilized antibiotic treatment as a means to promote bodily growth in both cattle and poultry [31,32]. Although antibiotic growth promoters have been met with considerable controversy given the concern for inducing population wide antimicrobial resistance [33], the mechanism behind the growth promotion is worthy of investigation. Some have postulated that this growth may be secondary to enhanced nutrient uptake in the setting of potential mucosal growth [31]. In fact, previous studies in broilers (chickens bred specifically for the purpose of meat production) treated with antibiotics have demonstrated enhancement in villus length and intestinal mucosal growth [34]. Similar clinical benefits have also been demonstrated in piglet models of necrotizing enterocolitis (NEC), where antibiotic therapy has been proven to be protective against damage to the intestinal mucosa [35]. More importantly, antibiotics have a demonstrated benefit in patients with short bowel syndrome with the intent of reducing the incidence and burden of small bowel bacterial overgrowth [36]. The present study may provide insight into some of the benefits of antibiotics in children with short bowel syndrome, and potentially the effect of antibiotic growth promoters in the livestock industry.

There are considerable data in the literature that suggest findings contradictory to our own [26,37–39]. In many instances, indigenous bacteria and probiotics have been demonstrated to promote intestinal epithelial proliferation [26,40,41]. Notably, our results demonstrate that a depleted intestinal microbiome has the potential to result in increased proliferative indices, as evidenced by the crypt proliferation index. We hypothesized that the increased villus height would be accompanied by increases in crypt proliferation and demonstrated this through intraperitoneal BrdU injection and subsequent immunostaining. While this was an intuitive finding to our team, others have reported the opposite effect, demonstrating decreased crypt proliferation in antibiotic treated mice and in germ-free mice, both in the small intestine and the colon [26,37]. Furthermore, others have suggested that the intestinal microbiome is essential for villus lacteal integrity and development and that treatment with broad spectrum antibiotics may even result in intestinal injury [38,39]. Inconsistencies between these findings and our own may be related to the specific antibiotic regimens utilized, the duration of antibiotic therapy, the age of experimental mice utilized, the region of small intestine analyzed, and the specific degree to which the microbiome was reduced or altered. Regardless, these findings shed light on some of the barriers that remain to utilizing the intestinal microbiome as a therapeutic target.

There are limitations in studying the small intestinal microbiome in a murine model when the ultimate goal is to generate clinically translatable data. Most importantly, the murine microbiome is not entirely representative of the human microbiome, with some reporting that up to 85% of the bacterial genera found in the murine microbiome are not present within the human microbiome [42]. Furthermore, the present study focuses on the presumed microbial alterations that occur within the small intestine, while the vast majority of established microbiome studies examine fecal samples and resultant effects on the colonic mucosa. There are few studies focused on investigating the small intestinal microbiome alone, which may be attributable to the relatively low abundance of microbial flora and the difficulty of obtaining samples both clinically and scientifically in *in vivo* experiments [43]. In addition, those that have studied this unique population of bacteria, suggest that the small intestinal microbiome is entirely distinct from the colonic microbiome and that of fecal samples [44]. Not only is the small intestinal microbiome unique from that of the colon, it also has been demonstrated to be more variable amongst individual subjects and may even fluctuate daily within a given subject [45]. Thus, while studies investigating the small intestinal microbiome may be more clinically relevant to understanding and treating small intestinal diseases, the procedures and techniques to successfully study this bacterial population clinically have yet to be fully developed. Given the reported microbial variability amongst subjects, the incongruity of composition between the human and murine microbial milieus, and the difficulty of feasibly collecting and measuring the composition of the small intestinal microbiome *in vivo*, the present study elected to instead focus on the output measure of mucosal adaptation rather than specific microbial alterations at the small intestinal level. We utilized the absolute quantification of fecal 16s rRNA copies as a surrogate to signify intestinal wide microbiome depletion. We did not specifically set out to identify particular species of bacteria that may result in mucosal growth, but rather we aimed to identify a potential pharmacologic therapy that would achieve the same result.

Concern regarding the toxicity and safety associated with using broad spectrum antibiotics led us to study the effects of a number of narrower spectrum antibiotic regimens all with a presumably lower side effect profile. Of the regimens trialed, Ampicillin/Vancomycin and Ampicillin/Vancomycin/Metronidazole appeared to result in considerable intestinal mucosal growth. While the intestinal mucosal response to these regimens was not quite as robust as the full broad spectrum antibiotic regimen, they present an intriguing area worthy of further study. Future studies should be designed to focus on tailoring a narrow antibiotic regimen with an appropriate side effect profile, limiting any off target physiologic harm to the host. Furthermore, such a regimen must be trialed in an animal model of short bowel syndrome, which has already undergone an adaptive mucosal response, prior to drawing any definitive conclusion on its effect in those recovering from short bowel syndrome. Finally, we postulate that there exists a molecular mechanism by which the microbiome either stimulates or inhibits enterocyte proliferation and thus small intestinal mucosal growth. Identification of this mechanism would allow more specific targeting of this pathway without the use of antibiotics.

In conclusion, we have shown that enteral administration of broad-spectrum antibiotics to mice with a conventional microbiome reduces the bacterial density of the intestinal microbiome and stimulates ordered and lasting small intestinal mucosal growth, resulting in taller villi with retention of normal cellular composition. The exact mechanism behind this observed mucosal growth remains unclear but is likely a direct result of depletion of the intestinal microbiome. Some species of indigenous bacteria are thought to be capable of producing metabolites that signal colonic enterochromaffin cells to synthesize serotonin (5-HT) [46]. Furthermore, enhancement of intestinal serotonin (5-HT) has been shown to result in small intestinal mucosal growth and enhanced mucosal absorptive capacity [4,47–49]. Given the demonstrated capabilities of 5-HT to promote mucosal growth and its known connection with the intestinal microbiome, it is plausible that alterations in gut bacteria can result in changes to mucosal growth factors and neurotransmitters potentially giving rise to structural changes within the mucosa that have been documented in this study. Further mechanistic studies are warranted and may provide guidance for the development of future therapies for patients with malabsorptive disorders.

## Materials and methods

### Animals

Conventional flora (CONV) C57Bl/6 mice were bred at The Jackson Laboratory (Farmington, CT) and transferred to Yale University. Germ-free (GF) C57Bl/6 mice were obtained from The Jackson laboratory (Farmington, CT), then bred and rederived germ-free at the Yale Microbial Sciences Institute (New Haven, CT). Mice were singly housed in either pathogen-free conditions (CONV) or within flexible plastic gnotobiotic isolators (GF) on a 12-hour dark/light cycle. Autoclaved cages and water bottles were exchanged weekly to minimize environmental bacterial overgrowth in pathogen free conditions. All mice were provided standard, autoclaved mouse chow *ad-libitum*. Prior to all experiments mice were age (10–13 weeks), weight, and sex matched. Both CONV and GF mice were divided into two treatment groups with *ad libitum* access to either an antibiotic solution (Ampicillin, Ciprofloxacin, Metronidazole, Vancomycin, Meropenem) mixed in artificial sweetener (n = 5CONV, 6GF) or artificial sweetener alone (n = 3CONV, 6GF) based upon previously reported studies [22,24,26]. Throughout all experiments, control mice were treated with a vehicle of artificial sweetener mixed in the drinking water. Metabolic measurements including mouse weight, food consumption (grams), and water consumption (milliliters) were obtained weekly. At the conclusion of the study period, mice were euthanized by CO2 asphyxiation and cervical dislocation.

### Antibiotic solution

An antibiotic regimen was adapted from previously reported regimens based on its ability to induce intestinal microbiome changes that substantially alter the host intestinal microbiome and mimic near germ-free conditions [22,24,26]. The solution consisted of deionized water with solubilized Ampicillin (100mg/L), Ciprofloxacin (200mg/L), Metronidazole (1g/L), Vancomycin (500mg/L), Meropenem (100mg/L), and grape flavored Kool-Aid (20mg/ml) to counteract the unpalatable effects of Metronidazole. The antibiotic solution was prepared fresh every 3–4 days. Mice in both treatment groups were restricted from accessing the standard central water supply to ensure near steady state antibiotic administration. All control mice were given a vehicle of Kool-Aid (20mg/ml) in deionized water. All mice underwent routine bolus of normal saline solution at treatment day 7 to prevent dehydration during the transition period to the antibiotic solution.

Narrower antibiotic regimens were also trialed in an effort to find a less toxic combination of antibiotics and to decrease the potential for antibiotic resistance in the microbiome. To do this, additional CONV mice were assigned to groups with either a limited antibiotic solution in a vehicle of artificial sweetener or artificial sweetener alone. Mice (n = 4 per group) were divided into four treatment groups: Vehicle, Ampicillin (A), Ampicillin/Vancomycin (AV), and Ampicillin/Vancomycin/Metronidazole (AVM).

The reversibility of the effects of an antibiotic regimen on the intestinal mucosa were also tested. To do this, CONV mice were allowed ad libitum access to either an antibiotic solution (Ampicillin, Ciprofloxacin, Metronidazole, Vancomycin, Meropenem) mixed in artificial sweetener (n = 15) or artificial sweetener alone (n = 12). After 14 days of treatment (Day 14), antibiotics were discontinued and all mice were transitioned to ad libitum artificial sweetener. Mice from both groups (antibiotic and vehicle) were then euthanized at varying time points for evaluation of the intestinal mucosa: Day 14 (n = 4,3), Day 28 (n = 4,3), Day 42 (n = 3,3), and Day 56 (4,3). Untreated CONV mice were utilized as day 0 controls (n = 2).

### Tissue procurement

All experimental mice underwent identical procedures for small intestinal procurement. At the conclusion of the study period, mice were euthanized by CO2 asphyxiation and cervical dislocation. A midline laparotomy incision was made and the small intestine identified from the ligament of Treitz to the ileocecal valve. First, the condition of the GI tract was assessed grossly. Then the entirety of the small intestine was harvested by sharp dissection, chilled in phosphate-buffered saline (PBS), and subsequently flushed with 10% neutral buffered formalin (NBF). The most distal 2cm of ileum and the most proximal 1cm of jejunum were discarded. 2cm segments of the proximal, middle, and distal small bowel were then isolated and fixed in 10% NBF for a minimum of 24 hours prior to paraffin embedding and mounting on slides for evaluation of morphometric parameters.

### Microbiome analysis

Fecal pellets were collected sterilely into Eppendorf tubes at experimental day 0, 14, and 28. Samples were flash frozen in dry ice and stored in -80°C for processing at a later date. DNA samples were extracted via Qiagen’s DNeasy PowerSoil Pro Kit (Qiagen). Samples were then quantified via qPCR using primers for the variable region 4 (515f/806r) of the 16S rRNA gene. The absolute quantification of the microbial sample was calculated as the total number of 16S copies per sample divided by sample weight in grams.

### Morphometric parameters

Paraffin sections of intestine from all treatment groups underwent standard hematoxylin & eosin (H&E) staining. Slides were examined under 100-200x utilizing standard brightfield microscopy (Axio Imager M1, Zeiss, OberKochen, Germany) and later analyzed using ImageJ-FIJI software (NIH, Bethesda, MD) to determine villus height (VH), villus width (VW), crypt depth (CD), and crypt width (CW). Villi were measured only when intact from crypt-villus junction to crypt-villus junction with a visible central lacteal. Crypts were measured only when intact from crypt-villus junction to crypt-villus junction with at least partial visualization of adjacent villi. A minimum of thirty villi and fifteen crypts were measured per animal. Mucosal surface area (MSA) was calculated by applying measured data to a previously defined mathematical formula [50]. The investigators measuring morphometric parameters were blinded to study group until measurements were completed and recorded.

### Citrulline assessment

Serum citrulline, a marker of functional enterocyte mass [51,52], was measured calorimetrically. Blood samples were obtained via standard retrobulbar technique on day 0, 14, and 28. Serum was then isolated from each sample and stored at -80C until further analysis. Serum citrulline levels were first quantified in duplicates using Citrulline Assay Kit (Cell Biolabs, San Diego, CA). Samples were ultimately analyzed in triplicates spectrophotometrically by measuring absorption at 560nm against standard citrulline curves.

### Proliferation

Crypt proliferation index (CPI) was calculated by quantifying bromodeoxyuridine (BrdU) staining of crypt cells. One hour prior to bowel harvest, mice received an intra-peritoneal injection of 100mg/kg bodyweight 5-Bromo-2’-deoxyuridine (BrdU; Alfa Aesar by Thermo Fisher Scientific, Haverhill, MA). BrdU incorporation was determined immunocytochemically with the BrdU in-Situ Detection Kit (BD Pharmingen, San Diego, CA). CPI was calculated as the number of BrdU-positive crypt cells as a fraction of the total number of cells per crypt and then converted to a percentage. Only intact crypts from crypt-villus junction to crypt-villus junction were included within the analysis. A minimum of thirty BrdU-stained crypts per animal were examined for analysis.

### Apoptosis

Degree of apoptosis was assessed using the terminal deoxynucleotidyl transferase dUTP nick end labeling (TUNEL) assay (In Situ Cell Death Detection Kit, Roche Diagnostics, Mannheim Germany). Positive TUNEL cells were detected under 400x using confocal microscopy (Axio Observer Z1, Zeiss OberKochen, Germany). TUNEL values were calculated as the total number of positive TUNEL cells per villus. A minimum of thirty villi per animal were examined for analysis.

### Cellular composition

Cellular composition of villi and crypts was investigated by standard immunofluorescent staining to identify and quantify enterochromaffin cells (ECC), goblet cells (GC), enterocytes (EC), and Paneth cells (PC) in villi and crypts. All immunofluorescent protocols were standardized throughout the experiment. In each case, unstained and mounted paraffin tissue sections were deparaffinized and rehydrated through a series of 3-minute xylene and EtOH washes (100% xylene, xylene 1:1 with 100% EtOH, 100% EtOH, 95% EtOH, 70% EtOH, 50% EtOH). The tissue was then submerged in 10mM sodium citrate buffer (pH 6) and heated for 20 minutes for antigen retrieval. After cooling and rinsing, the samples were permeabilized with 1% SDS and blocked with 5% bovine serum albumin (BSA) in PBST. The BSA was removed after a 1-hour incubation period and replaced with the following primary antibodies: ECC–ChrA mouse species 1:100 in 5% BSA (Santa Cruz Biotechnology Inc) and PC–lysozyme rabbit species 1:500 in 5% BSA (Novus Biologicals). Primary antibodies were incubated overnight in a humidified chamber at 4°C. The secondary antibodies were applied the following day: PC-Goat anti-mouse 488 1:200 (Life Technologies), ECC-Goat anti-rabbit 555 1:500 (Life Technologies). Following a 1-hour incubation period in an opaque humidified chamber, excess secondary antibody was washed away with a series of PBS washes. Slides were mounted with Prolong Gold 4’,6-diamidino-2-phenylindole (DAPI) (Life Technologies).

Immunofluorescent slides were examined under 400x confocal microscopy (Axio Observer Z1, Zeiss OberKochen, Germany) in order to determine the cellular composition of villi and crypts. ChrA-positive cells were counted as ECCs, lysozyme-positive cells were counted as PCs. The presence of GCs was determined by the characteristic goblet morphology and relative lack of autofluorescence. Total ECs were calculated by counting the total number of DAPI positive cells and subtracting the total number of ECC and GC. Enterochromaffin cells, goblet cells, and enterocytes were ultimately counted and calculated as a percentage of total cells per villus, whereas Paneth cells were counted and presented as a total number of cells per crypt.

### Statistical analysis

Statistical analysis was performed using GraphPad Prism version 8.0.0 for MAC OS X (GraphPad Software, La Jolla, CA). Means were compared with Student’s *t*-test to a significance of p<0.05.

### Study approval

Animal protocols were approved by Yale University’s Institutional Animal Care and Use Committee (IACUC #: 2016–11567).
